# Glycinergic Signaling in Macrophages and Its Application in Macrophage-Associated Diseases

**DOI:** 10.3389/fimmu.2021.762564

**Published:** 2021-10-05

**Authors:** Zhending Gan, Meiyu Zhang, Donghui Xie, Xiaoyan Wu, Changming Hong, Jian Fu, Lijuan Fan, Shengyi Wang, Sufang Han

**Affiliations:** ^1^ College of Animal Science, South China Agricultural University, Guangzhou, China; ^2^ College of Animal Science and Technology, Guangdong Polytechnic of Science and Trade, Guangzhou, China; ^3^ Nanchang Academy of Agricultural Sciences, Nanchang, China; ^4^ Key Laboratory of Veterinary Pharmaceutical Development, Ministry of Agricultural and Rural Affairs, Lanzhou Institute of Husbandry and Pharmaceutical Science, Chinese Academy of Agricultural Science, Lanzhou, China

**Keywords:** glycine, macrophage, NF-κB, miRNA, inflammation

## Abstract

Accumulating evidences support that amino acids direct the fate decision of immune cells. Glycine is a simple structural amino acid acting as an inhibitory neurotransmitter. Besides, glycine receptors as well as glycine transporters are found in macrophages, indicating that glycine alters the functions of macrophages besides as an inhibitory neurotransmitter. Mechanistically, glycine shapes macrophage polarization *via* cellular signaling pathways (e.g., NF-κB, NRF2, and Akt) and microRNAs. Moreover, glycine has beneficial effects in preventing and/or treating macrophage-associated diseases such as colitis, NAFLD and ischemia-reperfusion injury. Collectively, this review highlights the conceivable role of glycinergic signaling for macrophage polarization and indicates the potential application of glycine supplementation as an adjuvant therapy in macrophage-associated diseases.

## Introduction

Macrophages are found in almost all tissues such as Kupffer cells in hepatocyte (1) and microglia in central nervous system (2). These macrophages engulf cellular debris, microbes, death cells and foreign substances by stretching filopodia (3, 4). Although the polarizations of macrophages are multiple, they are roughly polarized to two distinct subsets: classically activated (M1) phenotype and alternatively activated (M2) phenotype (5, 6). Macrophages polarize into M1 phenotype to perform their pathogen-scavenging function when exposed to T-helper 1 (Th 1) type cytokines or inflammatory mediators, such as interferon gamma (IFN-γ) and lipopolysaccharide (LPS) (7), or M2 phenotype to perform their anti-inflammatory effects, including wound healing and anti-tumor ability under conditions of exposure to Th 2 cytokines like IL-4 and IL-10 (8). Indeed, various contributors are related to the fate of macrophages. Notably, metabolism pathways and metabolites are the best examples for directing macrophage growth and survival by providing energy and substrates, and instructing functions of macrophages (9, 10). For example, altered amino acid metabolism [e.g., arginine metabolism (11)] is a well-accepted character to define macrophage polarization.

Traditionally, amino acids are simply divided into two categories: essential amino acids and non-essential amino acids (12). However, many traditionally considered non-essential amino acids are not only used as substrates for protein and peptide synthesis, but also involved in regulating metabolism, signal transduction and immune responses (13). Glycine consists of one carbon (C) atom, two hydrogen (H) atom, one carboxyl-group (COOH) and one amino-group (NH_2_) (14). Of note, recent studies have shown that glycine affects functions of macrophage (15, 16). In this review, we will summarize glycinergic system in macrophages, discuss how glycine contributes to the polarization of macrophages, and list some examples that glycine mediates macrophage-associated diseases.

## Glycinergic System in Macrophages

### Glycine Receptors in Macrophages

Glycine is an inhibitory neurotransmitter (17), which exerts inhibitory effect by binding to glycine receptors (GlyRs) (18–20). GlyRs consist of α subunits (48kDa), β subunits (58kDa) and a 93 kDa subunit anchoring protein gephyrin (21). GlyRs also present in non-neuron cell membrane, such as macrophages (20, 22). For example, the subunits of GlyRs are found in rat Kupffer cells, splenic macrophages and alveolar macrophages, and the sequences of the cloned fragment for the GlyRs β subunit in macrophages are more than 95% homologous with the GlyRs from the spinal cord (22). It should be noted that the GlyRs subunits differ in various types of macrophages. For example, Kupffer cells have α1-subunit, α4-subunit and β-subunit, while α2-subunit, α4-subunit and β-subunit are found in splenic and alveolar macrophages, as well as only α1 subunit in the peritoneal macrophages in rats (22, 23). The reasons for these differences might result from the origins of macrophages (24) (embryonic origin and monocyte derivation), species of animals and even culture condition of isolated macrophages. It is also intriguing to know whether such difference presents in mouse or human macrophages. Although GlyRs have been identified on macrophages, no studies have investigated the effects of GlyRs subunits in macrophage fate decision. Notably, blocking the receptor with strychnine (25, 26) alleviates glycine-induced intracellular Ca^2+^ decrease in LPS-stimulated macrophages (25, 27, 28), suggesting the receptor highly shapes the fate decision of macrophages. To fully illustrate the function of GlyRs in macrophages, the comparative analysis towards expression and location of GlyRs in macrophages from different tissues and subsets (e.g., resting macrophages *vs.* M1 phenotype or M2 phenotype) should be performed. Then the function of GlyRs subunits in macrophage fate decision can be explored with chemical ablation or genetic manipulation.

### Glycine Transporters in Macrophages

In the central nervous system (CNS), glycine is transported into cells by neutral-amino-acid transporters (NAATs, **Table 1**) (29, 30); however, the presence of NAATs in macrophages remains to fully explore. Interestingly, rat M1 macrophages are sensitive to NAATs substrate 2-aminoisobutyric acid (AIB) (23) and the application of methylamino-AIB inhibits glycine-induced inward currents in microglia (31), suggesting that NAATs might be expressed in macrophages. As expected, it has been demonstrated that rat peritoneal macrophages express at least one of NAATs, especially glycine transporter-1 (GlyT1) (23). Further investigations are needed to examine the expression of NAATs in mice and human macrophages.

**Table 1:** Neutral-amino-acid transporters which transport glycine.

System	Gene	Transporters (Full name and abbreviation)
**Sodium dependent NAATs**		
**A**	SLC38A1	Serine acetyltransferase 1 (SAT1)
	SLC38A2	SAT2
	SLC38A4	SAT3
**Gly**	SLC6A9	Glycine transporter 1 (GlyT1)
	SLC6A5	GlyT2
**Sodium independent NAATs**		
**asc**	SLC7A10	Asc Type Amino Acid Transporter 1/2 (ASC1/2)
**imino**	SLC36A1	Proton-coupled amino acid transporter 1 (PAT1)
	SLC36A2	Proton-coupled amino acid transporter 2 (PAT2)

## Glycine Metabolism in Macrophages

In mammals, glycine can be synthesized from serine, choline, threonine and hydroxyproline by different metabolic pathways (32). Since serine and glycine are biosynthetically linked (33), serine and its precursors can generate glycine. The conversion of serine to glycine catalyzed by serine hydroxymethyltransferase (SHMT) is the main way for glycine synthesis (34, 35). When glycine deficiency occurs, such as intrinsic glycine uptake capacity limitation or environmental glycine deprivation, SHMT can support glycine synthesis (36).

In addition to participating in protein synthesis, glycine is a precursor of peptides, nucleic acids as well as methyl donors. Upon LPS stimulation, the levels of intracellular glycine and glycine metabolites such as glutathione (GSH) and S-adenosylmethionine (SAM) increased (37–39). Interestingly, adding glycine to the serine-deprived medium failed to rescue IL-1β secretion in macrophages upon LPS stimulation (38). Besides this, lack of glycine cannot affect the polarization of macrophages (39). Thus, extracellular glycine may not influence macrophage metabolism. U-[13C]-labeling shows that glycine is mainly converted from glucose and serine, and it can be subsequently converted to ADP, ATP, GSH and SAM (38). Strikingly, U-[13C]-glycine revealed a remarkable attenuation of extracellular glycine-derived GSH compared to serine (synthesis from glycine)-derived GSH (38). Moreover, supplementary glycine in serine deprived medium failed to rescue intracellular GSH in macrophage. These phenomena indicate that glycine utilization in macrophages is mainly through intracellular conversion of serine, not *via* exogenous glycine supply.

## Glycine Regulates Signaling Pathways in Macrophages

The functions of macrophages are highly responsive to their micro-environmental stimuli. Upon the activation of Toll-like receptor (TLR) or interferon signaling, M1 macrophages arise in inflammatory to eliminate pathogens (40–42). Whereas M2 macrophages, usually found in Th2-dominated responses, can mediate helminth immunity, asthma, and allergy (43).

**Figure 1:**
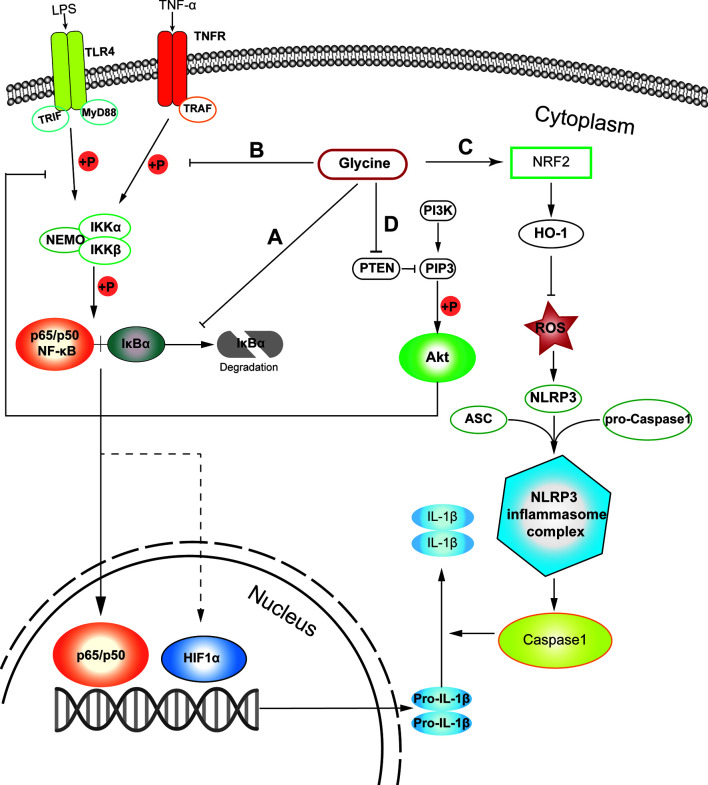
Probable cellular pathways that glycine influences M1 macrophages polarization. **(A)** Glycine inhibits the degradation of IκB in M1-macrophages. **(B)** Glycine inhibits M1-macrophages polarization *via* inhibiting IKK phosphorylation. **(C)** Glycine up-regulates NRF-2/HO-1 to blunt NLRP3 in inflammasome in M1-macrophages. **(D)** Glycine inhibits NF-κB by blocking PTEN to up-regulate Akt in M1-macrophages. LPS, lipopolysaccharide; TLR4, toll-like receptor 4; MyD88, myeloid differentiation primary response gene 88; NF-κB, nuclear factor kappa-light-chain-enhancer of activated B cells; IκB, inhibitor of NF-κB; IKK, IκB kinase; TNF-α, tumor necrosis alpha; TNFR, TNF-α receptor; TRAF, TNFR associated factor; PTEN, phosphatase and tensin homolog deleted on chromosome ten; PIP3, phosphatidylinositol (3,4,5)-trisphosphate; Akt, protein kinase B.

Among various signaling pathways regulating macrophage inflammation, NF-κB is a main contributor to orchestrate macrophage polarization (44). Glycine can prevent the activation of nuclear factor-κB (NF-κB) by inhibiting the degradation of inhibitor of NF-κB (IκB) in pro-inflammatory macrophages (**Figure 1A**) (45). Additionally, glycine affects inflammasome assembly in pro-inflammatory macrophages (46). However, given glycine treatment could induce IκB degradation in resting macrophages (45), we still could not exclude the possibility that glycine causes stress responses in resting macrophages. In addition, in the context of glycine treatment, the decreased phosphorylation of IκB kinase-α (IKK-α) and IκB kinase-β (IKK-β) is also observed (45, 46) (**Figure 1B**). Glycine reduces LPS-induced upregulation of nucleotide binding domain like receptor protein 3 (NLRP3) (47). This process can be achieved by up-regulating the expression of NRF2 and its down-stream signaling pathways to eliminate reactive oxygen species (ROS) (47) (**Figure 1C**).

PI3K (phosphatidylinositol 3-kinase) and Akt (protein kinase B) pathways regulate tremendous signaling pathways, including NF-κB and mitogen-activated protein kinase (MAPK) signaling (48) related to macrophage polarization (49). Glycine can up-regulate Akt by blocking phosphatase and tensin homolog deleted on chromosome ten (PTEN), then inhibit NF-κB and hypoxia induced factor-1 α (HIF1-α) in microglia (50) in the context of ischemia-reperfusion injury. Except for macrophages, glycine also inhibits PTEN and activates Akt in other tissues or cells (51, 52) (**Figure 1D**). Unfortunately, there is still no direct evidence showing whether glycine can affect proinflammatory macrophage polarization induced by canonical stimuli (e.g., LPS and/or IFN-γ) through PTEN-Akt pathway. Notably, Akt kinases have distinct effects in macrophage polarization, with Akt1 ablation leading to an M1 phenotype and Akt2 ablation resulting in an M2 phenotype (53). It has not been studied which subunit of Akt is regulated by glycine. Therefore, it is necessary to further explore the connection between glycine and the Akt signaling pathway in guiding macrophages polarization.

## Glycine Alters microRNAs in Macrophages

**Figure 2:**
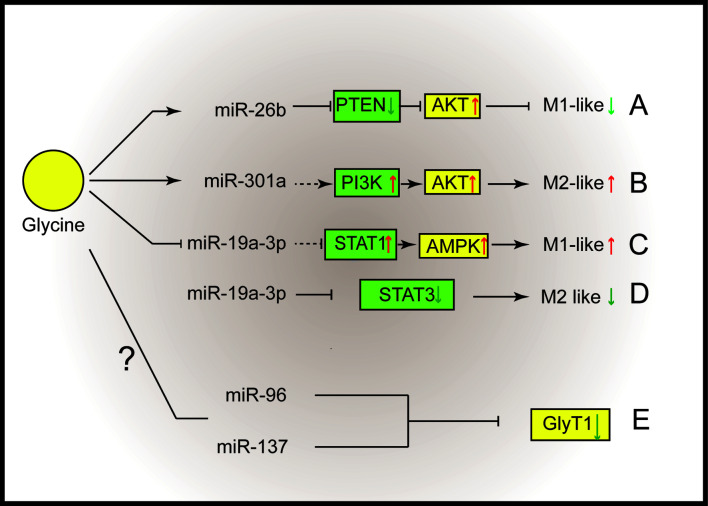
Glycine shapes macrophage polarization through micro-RNAs. **(A)** Glycine up-regulates miR-26b to blunt M1-microglia polarization by suppressing PTEN and activating Akt. **(B)** Glycine possibly up-regulates miR-301a to promote M2-macrophages polarization *via* activating PI3K/Akt. **(C, D)** Glycine down-regulates miR-19a-3p. **(C)** MiR-19a-3p negatively control STAT1 and AMPK to blunt M1-macrophages polarization. **(D)** MiR-19a-3p negatively control STAT3 to inhibit M2 macrophage polarization. **(E)** MiR-96 and miR-137 negatively regulate GlyT1. PTEN, phosphatase and tensin homolog deleted on chromosome ten; Akt, protein kinase B; STAT, signal transducer and activator of transcription; GlyT, glycine transporter.

MicroRNAs (miRNAs) play vital roles in a great deal of biological processes (54) and could function as crucial regulators that support macrophage polarization (54, 55). It has been reported that some miRNAs which associated with macrophages are related with glycine. For example, glycine alleviates subarachnoid-hemorrhage (SAH) induced neuron inflammation, which is mediated by miRNA-26b/PTEN/Akt signaling pathway in microglia (56) (**Figure 2A**). Inhibition of miRNA-26b or activation of PTEN expression suppressed the protective function of glycine (56). MiR-301a is abundantly expressed in hypoxic pancreatic cancer cell-derived exosomes (57, 58), which can promote M2 macrophage polarization through activating PTEN/PI3K signaling pathway (57). Interestingly, glycine has been reported to enhance the expression of miR-301a in the cortical neurons (59). Thus, miR-301a might be a potential target for glycine to regulate M2 macrophage functions (**Figure 2B**).

MiR-19a-3p can suppress LPS/IFN-γ-induced M1 macrophage polarization *via* inhibiting STAT1 (signal transducer and activator of transcription-1) (60). In addition, glycine regulates miR-19a-3p/AMPK pathway to alleviate ischemic stroke injury (61). Therefore, glycine may promote M1 macrophage polarization by regulating miR-19a-3p (**Figure 2C**). Besides influencing M1 macrophages polarization, miR-19a-3p is capable of suppressing M2 macrophage polarization by inhibiting STAT3 when overexpressed (62) (**Figure 2D**).

Notably, miRNAs can regulate GlyTs function. Human GlyT1 possesses several miRNAs targeting sites within the 3’UTR (miR-7, miR-30, miR-96, miR-137, miR-141). Among them, miR-96 and miR-137 negatively regulate GlyT1 under physiological conditions (63) (**Figure 2E**). It is intriguing to investigate whether microRNAs mediate the regulation of glycinergic system in macrophage polarization.

## Application of Glycine in Macrophage-Related Diseases

### Obesity and Associated Metabolic Diseases

The white adipose tissue can produce many adipokines such as leptin, TNF-α, and interleukins, due to the accumulation of macrophages (64–66). In adipocytes differentiated 3T3-L1 cells, applying 10 mM glycine in the medium decreases the expression of IL-6, resistin and TNF-α (67). Similarly, in glutamate-induced obese mice, the application of glycine reprograms fat metabolism and decreases the expression level of TNF-α and IL-6 (68). Serum and liver glycine levels in obese rats are lower than thin rats (69) and dietary supplementation with glycine lowers circulating triglycerides in Zucker fatty rats (70). These phenomena were also found in humans. The plasma glycine level is lower in obese and diabetic patients (71, 72) in comparison to healthy donor. In clinical application, dietary supplementation of glycine can improve insulin response and glucose tolerance (73, 74). Impaired glycine metabolism may play a causative role in NAFLD, glycine-based treatment stimulating hepatic GSH synthesis in experimental NFLD (75). These results show that glycine could be helpful for alleviating inflammatory state in obesity.

Non-alcoholic steatohepatitis (NASH) and non-alcoholic fatty liver disease (NAFLD) are stubborn illnesses because of their prevalence, difficulties in diagnosis, complex pathogenesis, and lack of approved therapies (76). Macrophages are involved in the development of steatosis, inflammation and fibrosis in NASH (77). Furthermore, an increase of M1 macrophages in adipose tissue contribute to NASH due to its secretion of various proinflammatory signals, and these inflammatory factors move to hepatic and trigger local macrophages polarization (78). It has been found that glycine alleviates NASH index in high fat and high sucrose induced NASH in rats (79). Like obesity patients, plasma glycine levels are lower in NAFLD patients (80). Moreover, in a metabolic steatohepatitis mice model, glycine decreases cytokines level and increases M2/M1 macrophages ratio (81). These results indicate that glycine may have potential to treat non-alcoholic hepatic diseases.

Glycine could regulate the intestinal flora and decrease intestine macrophage infiltration in mice under LPS stimulation (82). Interestingly, pro-inflammatory macrophage accumulation was found in obesity humans (83). Besides this, increased pro-inflammatory macrophages were found in the gut of high fat diet (HFD) fed mice (84). Thus, glycine may potential to decrease intestinal pro-inflammatory macrophages infiltration to help alleviating obesity and obesity associated metabolic diseases. Whether glycine can affect intestinal macrophage by affecting intestinal flora needs to be further investigation.

### Ischemia-Reperfusion Injury

Ischemia-reperfusion injury is a serious problem after visceral transplantation (85, 86). Glycine significantly increases the survival rate after ischemia-reperfusion and alleviates the inflammatory injury from ischemia-reperfusion. Local perfusion with glycine can alleviate warm ischemia-reperfusion injury in small intestine of rats (87, 88) and liver of mice (89–91), as well as renal ischemia reperfusion injury caused by renal hypothermic (92). Interestingly, there exists a solid connection between ischemia-reperfusion injury and macrophages. The activation and migration of macrophages can aggravate inflammation, apoptosis or other stress in apparatus (93, 94). Fortunately, the researchers found that glycine inhibited the activation of Kupffer cells and their interleukins production during liver ischemia-reperfusion (89, 90, 95). In short, glycine is helpful for postoperative recovery after ischemia-reperfusion.

### Cancer/Tumor

Tumor associated macrophages (TAMs) are highly prevalent in many solid tumors (96, 97). Disrupting the malignant interaction between TAMs and cancer cells may greatly contribute to the survival of cancer patient. However, current targeted therapies of TAMs still fail to give a satisfied effect in tumor control because it is truly difficult to completely clear tumor and simultaneously avoid the high toxicity to patients. Thus, it is urgent to find effective and safe targeted TAM therapies.

Regulating TAMs is one of the targets for cancer treatment. Because of its infinite proliferation ability, cancer cells are highly dependent on glycine and serine uptake for nucleotide synthesis and one-carbon metabolism. Silencing SHMT2 and/or depriving extracellular glycine halts the rapid proliferation of cancer cells, but is not capable of blocking their proliferation completely (97). This phenomenon can rescue by the addition of glycine in the medium (97). Strikingly, glycine is generally consumed by highly proliferative cancer cells, but released by slow-proliferating cells (97). Thus, the demands of glycine may be distinct in different types or different proliferation states of cancer cells. Furthermore, high glycine concentration in tumor microenvironment can be consider as a clinical indicator of poor prognosis of tumor (98). Regulating glycine level in the tumor microenvironment may be an effective treatment for inhibiting the proliferation of cancer cells.

### Colitis

Colitis is an idiopathic intestinal inflammatory disease involving the colon, the clinical manifestations are diarrhea, abdominal pain, and even bloody stools (99, 100). Glycine altered colon microbiota and serum amino acids concentration, as well as colon interleukin level in 5% acetic acid induced colitis in mice (101). Similarly, dietary supplementation of 5% glycine alleviates colitis induced by 2,4,6-trinitrobenzene sulphonic acid (TNBS) and dextran sulfate sodium (DSS) in rats (102). Besides this, glycine supplementation ameliorates *C. redentium*- induced colitis and enhancing the abundance of *Lactobacillus (* 103). In summary, glycine supplementation may a nutritional strategy to alleviate colitis.

Taken together, these findings suggest that glycine has a certain preventive effect on macrophage-related diseases which are summarized in **Table 2**. However, the beneficial effects of glycine in other macrophage-associated diseases and the underlying mechanisms still need further investigation.

**Table 2:** Beneficial effects of glycine in other macrophage-associated diseases.

Model	Dose	Features	References
**Arthritis (Rat)**	Dietary supplementation with 5% glycine	Pro-inflammatory cytokines ↓	(104, 105)
**Acute pancreatitis (AP) (Rat)**	Intravenous injection of 100/300 mmol glycine	Pathological structure ↑; Pro-inflammatory cytokines ↓	(106)
		MPO activity ↓
**Oral gingival inflammation (Cultured gingival epithelial cells)**	5mM glycine supplemented in culture medium	Pro-inflammatory interleukin level ↓	(107)
		Nf-κB activation ↓	
**Endotoxin (LPS) shock (Rat)**	Dietary supplementation with 5% glycine	Survival rate ↑,	(108)
		Serum pro-inflammatory cytokines level ↓	
**Colitis (Rat and mice)**	Dietary supplementation with 5% glycine	Macroscopic and histologic scores ↑	(101, 102)

↑, increase/up-regulate; ↓, decrease/down-regulate.

## Concluding Remarks

In this review, we introduced glycinergic system in macrophages, and summarized how glycine shapes macrophages polarization. For glycinergic system, GlyRs could be found in macrophages, and the subunits of GlyRs are varied in macrophages with different origins. Though it has been already noted that NAATs exist in macrophages, it is not clear which type of NAATs is expressed in macrophages. Glycine is supposed to affect macrophage through different contributors. Mechanistically, glycine alters macrophage signaling pathways (e.g., NF-κB, NRF2, and Akt) and miRNAs. Interestingly, other signaling pathways [e.g., ERK (109)] might also mediate the functions of glycine. Therefore, it is not surprising that glycine could influence the progresses of several macrophage-associated diseases (e.g., colitis and NAFLD).

Indeed, the influences of glycine in macrophage activation are still worth further investigation. Firstly, it is not clear whether glycine can affect methylation reaction in macrophages. In one-carbon metabolism, glycine partly provides the carbon backbones required for the generation of SAM (110), which is the main methyl donor for cellular methylation reaction (39, 111). Recent studies have shown that the methylation of histone (39), DNA (112) or mRNA (113, 114) is closely related to macrophage polarization. Therefore, glycine is likely to affect macrophage polarization through methylation modification. Secondly, there are few studies on the effect of glycine on the metabolism of macrophages. Macrophage metabolism is highly related with the function output of macrophages (54). Considering glycine could impact HIF-1α and mTORC1 that are related to cellular metabolism (e.g., glycolysis), thus studying the effect of glycine on macrophage metabolism is meaningful to reveal the working mechanism of glycine on macrophages function. Finally, studying the effect of glycine on macrophages in the tumor microenvironment may reveal a potential target for cancer therapy. Therefore, it is necessary to find out the relationship between glycine, macrophage function and cancer progression.

## Author Contributions

ZG wrote the review article. ZG, SH, and XW revised the review article. CH, JF, and LF helped with designing figures and finding relevant literatures. MZ and DX reviewed and revised the grammar error in the manuscript. All authors contributed to the article and approved the submitted version.

## Funding

This study was supported by the Guangdong Basic and Applied Basic Research Foundation (2019B1515210002) and National Natural Science Foundation of China (31922079).

## Conflict of Interest

The authors declare that the research was conducted in the absence of any commercial or financial relationships that could be construed as a potential conflict of interest.

## Publisher’s Note

All claims expressed in this article are solely those of the authors and do not necessarily represent those of their affiliated organizations, or those of the publisher, the editors and the reviewers. Any product that may be evaluated in this article, or claim that may be made by its manufacturer, is not guaranteed or endorsed by the publisher.
